# Active Breaks: A Pilot and Feasibility Study to Evaluate the Effectiveness of Physical Activity Levels in a School Based Intervention in an Italian Primary School

**DOI:** 10.3390/ijerph17124351

**Published:** 2020-06-17

**Authors:** Alice Masini, Sofia Marini, Erica Leoni, Giovanni Lorusso, Stefania Toselli, Alessia Tessari, Andrea Ceciliani, Laura Dallolio

**Affiliations:** 1 Department of Biomedical and Neuromotor Science, University of Bologna, Bologna Via San Giacomo, 12, 40126 Bologna, Italy; alice.masini7@unibo.it (A.M.); erica.leoni@unibo.it (E.L.); giovanni.lorusso@unibo.it (G.L.); laura.dallolio@unibo.it (L.D.); 2 Department of Life Quality Studies, University of Bologna, Campus of Rimini, Rimini Corso d’Augusto 237, 47921 Rimini, Italy; andrea.ceciliani@unibo.it; 3 Department of Biomedical and Neuromotor Science, University of Bologna, Bologna Via Selmi, 3, 40126 Bologna, Italy; stefania.toselli@unibo.it; 4 Department of Psychology, University of Bologna, Bologna Viale Berti Pichat, 5, 40126 Bologna, Italy; alessia.tessari@unibo.it

**Keywords:** children, school based intervention, moderate to vigorous physical activity, accelerometers

## Abstract

*Background:* The school gives access to children, regardless of age, ethnicity, gender and socio-economic class and can be identified as the key environment in which to promote children’s physical activity (PA). The guidelines of the European Union recommend accumulating at least 10-min bouts of PA to reach the daily 60 min. Active breaks (ABs) led by teachers inside the classroom represent a good strategy to promote PA. The aim of this pilot and feasibility study was to evaluate the feasibility and effectiveness in terms of PA level of an AB programme in children aged 8–9 years attending primary school. *Methods:* A pre-post quasi-experimental pilot and feasibility study was performed in two primary school classes, one of which was assigned to a 14-week AB intervention (AB group) and the other to the control group (CG). At baseline and at follow-up, children were monitored for sedentary and motor activity during an entire week using ActiGraph Accelerometer (ActiLife6 wGT3X-BT). The satisfaction of children and teachers was assessed by self-administered questionnaires. *Results:* In the pre-post comparison, AB group (*n* = 16) showed a reduction in the minutes spent in weekly sedentary activity (−168.7 min, *p* > 0.05), an increase in the number of step counts (+14,026.9, *p* < 0.05) and in time spent in moderate to vigorous PA (MVPA): weekly MVPA: +64.4 min, daily MVPA: +8.05 min, percentage of MVPA: +0.70%. On the contrary, CG showed a worsening in all variables. ANCOVA analysis, after adjusting for baseline values, showed significant differences between the AB group and CG for time spent in MVPA, percentage of MVPA and step counts. The satisfaction of children and teachers was good. Teachers were able to adapt the AB protocol to the needs of the school curriculum, thus confirming the feasibility of the AB programme. *Conclusions:* This pilot and feasibility study showed the feasibility and effectiveness of the AB protocol and represented the basis for a future controlled trial.

## 1. Introduction

Scientific evidence continues to support the importance of physical activity (PA) for disease prevention and health promotion in children and youth [1]. On the contrary, sedentary behaviours, defined as any waking behaviours characterized by an energy expenditure ≤1.5 metabolic equivalents (METs; 1 MET = rest), both in sitting and lying posture, [2] may have detrimental health consequences during youth and later in life, depending on the type of sedentary behaviour and the age group studied [3]. Based on a systematic review, Cliff et al. (2016) stated that, while the evidence of a negative association between objectively measured sedentary time and health outcomes is still inconsistent, there is strong evidence that screen time is associated with negative health-related outcomes in children [4].

To reduce the risk of metabolic and cardiovascular diseases and to achieve all the benefits mentioned above, the World Health Organization (WHO) recommends that children and adolescents aged 5 to 17 years should accumulate at least 60 min per day of moderate to vigorous PA (MVPA) [5]. These guidelines are shared at the European level, except for Germany, and are also followed by Russia [6].

In the USA, approximately 24% of children aged 6–17 participate in 60 min of PA. In New South Wales children, this percentage is estimated at 19% [7]. Regarding the European countries, the percentage compliance to recommendations is generally low, and in many European Union countries, the number of inactive children (not compliant to recommendations) is constantly growing, particularly in Italy, where only 9.5% of boys and 2.6% of girls accomplish the daily requirement [8].

The school represents the environment in which children and adolescents spend most of their time; moreover, it gives access to children regardless of age, ethnicity, gender and socio-economic class. For these reasons, the school can be identified as the key environment and a powerful socializing setting in which to promote children’s good habits and PA [9]. The specific role of the school in promoting health behaviour was investigated by various studies, which analysed how different school organization could affect the adoption of good practices and avoid risk factors for health, also during adulthood [10,11,12]. In particular, PA promotion in the school setting could be a good strategy aimed at contrasting sedentary behaviours and improving physical skills and fitness.

Although physical education (PE) in the Italian setting is a fundamental part of all grade school curricula, it is often not adequately conducted, especially in primary schools, where the time devoted to PE is highly variable and lessons are performed by generalist teachers [13,14,15]. Consequently, the experience of PA in Italian children is frequently confined to participation in a few training sessions of sport alone, outside the school context, which is not enough to ensure the daily requirement of MVPA [16,17].

The guidelines of the European Union recommend that the full dose of 60 min can be accumulated in bouts of at least 10 min of PA [18]. The Italian Ministry of Health suggests, through its recent guidelines, to use innovative learning theories and a new perception of physical education, in which PA is promoted across various school-based activities [19]. In order to meet this goal and help children to be active, opportunities need to be provided outside the traditional occasions of motor activity (recess time and physical education class). Various studies were performed in the school setting to evaluate the potential benefits of classroom-based PA interventions [17,20,21]. Incorporating short bouts of activity throughout the school day could be a good strategy for children to accumulate the required amount of PA [22]. Moreover, short duration active breaks (ABs), led by trained teachers inside the classroom, are emerging as a promising way of increasing the PA levels and reaching positive learning outcomes [23].

A recent systematic review with meta-analysis suggests that ABs have positive effects in term of increasing PA levels and classroom behaviour. Lasting effects were obtained with the most intense (10 min thrice a day for 12 weeks) or longer (10−15 min once a day for 9 months) interventions. The majority of studies included in the review were performed in the USA and Australia, and to a lesser extent, in Europe [24]. Only one study about active breaks was carried out in Italy [25], which experimented an AB intervention, consisting of two daily PA breaks three times a week in a primary school, and showed the feasibility of the programme and its potential on the reduction of children’s inactivity. However, its results were not conclusive, due to the absence of a control group. The limited data available makes it necessary to investigate the Italian setting further. Therefore, in preparation for a future controlled trial, we conducted a study to pilot-test the effects of active breaks on physical activity levels in children attending a primary school in Northern Italy, and to evaluate children’s and teacher’s acceptability/satisfaction and, based on their feedback, the feasibility of the intervention.

We hypothesize that a 14-week classroom active break programme could positively affect the level of physical activity and could be a sustainable and feasible intervention carried out by teachers.

## 2. Materials and Methods

### 2.1. Study Design, Participants and Setting

We conducted a pre-post quasi-experimental pilot and feasibility study in a primary school of Northern Italy (Istituto Comprensivo “Castelletto”, Province of Bologna, Emilia-Romagna Region), from February 2019 to June 2019. The University of Bologna Bioethics Committee approved the study on the 25th January 2019. The study was carried out according to the Declaration of Helsinki and approved by the school board. First of all, we scheduled a preliminary meeting, where we informed the school manager and teachers about study aims, procedures and duration. After this step, a general meeting with the children’s parents was scheduled in order to explain the content and the context of the project. The informed consent of parents and permission for personal data processing were necessary to participate in the study.

### 2.2. Procedures

Based on the teachers’ willingness, two out of ten classes of the primary school participated in the study. One class of third grade was assigned to the experimental group (AB group) and another of the fourth-grade class to the control group (CG). The two teachers of the experimental class participated in a training day to understand all the exercises involved in the AB protocol. Control class teachers did not have to participate in any training. The children in the control group were only involved in pre-post measurements. The researchers provided a detailed manual with all the exercises proposed to the experimental group. The AB protocol was developed on the basis of previous literature investigated through a systematic review [24], with an innovative aspect represented by the introduction of high-intensity interval training (HIIT), consisting of 40” of vigorous PA alternated with 20” of recovery, performed at least once a day. The experimental class performed the AB protocol twice a day, usually the first break in the morning and the second in the afternoon, for all the weekdays, during an intervention period of 14 weeks. Both groups participated in the curricular physical education consisting of 2 h per week in the gym. Each AB lasted 10 min, divided into three different parts (Table 1).

In order to encourage the involvement of both teachers and children, the teachers were let free to implement some exercises of the AB programme by using curricular contents (i.e., music, English language, math content). They were asked to let us know comments and suggestions, especially regarding the organization of the classroom environment where the AB protocol was performed.

### 2.3. Measures

Baseline data were collected, both in the experimental and control groups, during the three weeks before the intervention. At baseline, weight, height, and waist circumferences of all children were measured, and body mass index (BMI) and waist/height ratio (WtHR) were calculated.

The time spent in physical activity and sedentary behaviour was monitored through Actigraph accelerometers (Actigraph, LLC, Pensacola, FL, USA) (ActiLife6 wGT3X-BT set to 10-s epochs). This instrument is reliable, valid and accurate, as supported by various pieces of evidence, especially in children [26,27,28,29]. The accelerometer data were analysed through ActiLife 6.13.3 software (ActiGraph, LCC, Pensacola, FL, USA). The activity levels were categorized using Evenson 2008: sedentary cut points (0–100), light (101–2295), moderate (2296–4011), vigorous (>4012), and MVPA minimum count (2296) [30,31,32].

The children were asked to wear the accelerometers over seven days (five weekdays and two weekend days), only to be removed when bathing, swimming and showering. Accelerometers were attached to an elastic belt around the waist. In accordance with the existing literature [33], we included in the analysis only children complying with some specific inclusion criteria: having worn the accelerometer on at least 3 weekdays and 1 weekend day, and for at least 10 h every day. After the end of the 14-week intervention, the same measurements and procedures were applied for the follow-up session. During both the baseline and follow-up evaluation, the intervention group did not perform the AB protocol.

We designed a self-administrated Active Break Questionnaire to investigate several aspects related to the feasibility of the programme. Once the intervention had finished, both the children and the two teachers of the experimental group completed the AB questionnaire. The children’s questionnaire included 5 items focused on the satisfaction and enjoyment in performing ABs, with a response scale of 3 qualitative points (yes, yes/no; no). The questionnaires for teachers included various domains regarding the level of satisfaction, the feasibility, the effectiveness and the managing of the ABs. It included 18 items exploring potential changes in the time on task classroom behaviour, attention and wellbeing of the children, and also their personal attitude in managing, implementing and organizing ABs. Teachers were asked to give a score from 1 to 5 for each question.

### 2.4. Statistical Analysis

Differences in Actigraph parameters from baseline to follow-up were analysed within groups, using the paired-samples t-test. Using anthropometric measures, children were stratified into two categories, both for WtHR (the value of 0.5 was chosen as cut-off of cardiovascular risk) [34,35,36] and BMI (overweight/obese and normal-weight children according to the International Obesity Task Force classification) [37]. Data were analysed by ANCOVA adjusting for baseline values, in order to evaluate the time spent in MVPA in function of WtHR and BMI. All tests were two-tailed, and the significance level was set to *p* < 0.05. All analyses were carried out using IBM SPSS Statistics version 20.0 (IBM, Armonk, NY, USA).

## 3. Results

### 3.1. Study Participants

**Figure 1:**
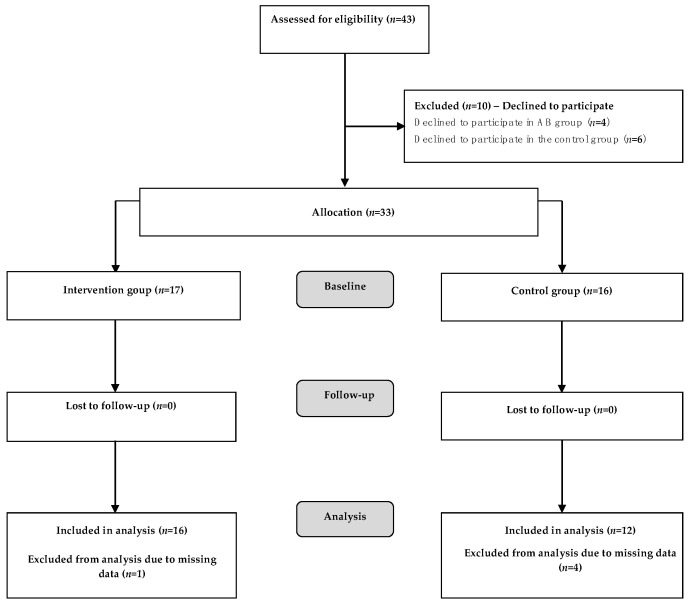
Flowchart of participants through each stage of the study.

The two classes participating in the pilot and feasibility study included a total of 43 children (Figure 1). Ten of these did not receive parents’ consent, so that they declined to participate. Thus, the number of participants at baseline was 33 (mean age 9.02 ± 0.11; males: 51.5%), 17 in the class assigned to the experimental group (AB group) and 16 in the class assigned to the control group (CG). The percentage of children with a WtHR ≥ 0.5 was 53.1%, while 55.2% were overweight or obese. The percentage of BMI overweight/obese in the control group was 64.3% vs. AB group 46.7%, the percentage of WtHR at risk (≥0.5) in the control group was 68.8% vs. AB group 37.5%. However, the differences between the groups are not statistically significant. The final analysis was performed on 16 children in the AB group and 12 children in the CG.

### 3.2. Physical Activity Levels

A total sample of 28 children provided baseline and follow-up measures that showed a normal distribution using the “Explore” function of SPSS (Statistical Package for Social Science). Overall, in the AB group, all Actigraph measures improved at follow-up, while in the CG, all parameters worsened (Table 2). In the AB group, a reduction in the number of minutes spent in sedentary activity was observed (−168.7 min). Consequently, the weekly and daily minutes spent in MVPA (Weekly MVPA +64.4 min, Daily MVPA +8.05 min), and the percentage of time spent in MVPA (+0.70%) increased in the AB group. There was also an improvement in the number of weekly step counts (+14,026.9), the only variable showing significant pre-post differences in the comparison within the AB group (*p* < 0.001). The CG showed a not significant statistically decrease in all the Actigraph parameters, from baseline to follow-up.

Using ANCOVA analysis, after adjusting for baseline values, a significant difference between the AB group and the CG was found in all variables, except the time spent in sedentary activities and daily MVPA (Table 2).

**Figure 2:**
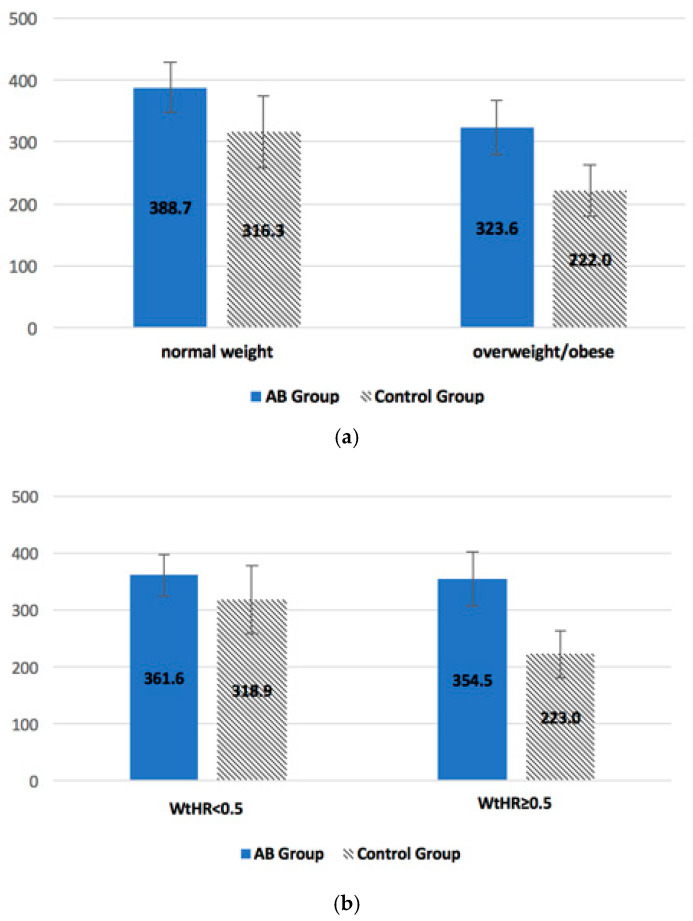
Total time (minutes) spent in weekly moderate to vigorous physical activity (MVPA) during the follow-up in relation to body mass index (BMI) categories (**a**) and waist-to-hip ratio (WtHR) categories (**b**) in AB group and control group (CG), adjusting for baseline values.

Stratifying children by BMI categories, it can be noted that, after the intervention, the time spent in MVPA is longer in normal-weight children compared with overweight/obese children, in both the AB group and CG (Figure 2a). However, no significant differences were found by ANCOVA, adjusting for baseline values. Stratifying children by WtHR, it can be noted that the time spent in MVPA is the same in all children of the AB group, while the CG children with WtHR ≥ 0.5 (cardiovascular risk) spent a shorter time in MVPA compared with children with WtHR < 0.5. Therefore, in both the risk and no risk categories of the AB group, a general improvement in the total time spent in MVPA was observed, while in the CG, the subgroup at risk (WtHR ≥ 0.5) showed a worsening compared with the subgroup not at risk (Figure 2b). Moreover, in this case, ANCOVA after adjusting for baseline values, did not highlight any significant differences between the AB group and CG.

### 3.3. Questionnaire Results

**Figure 3:**
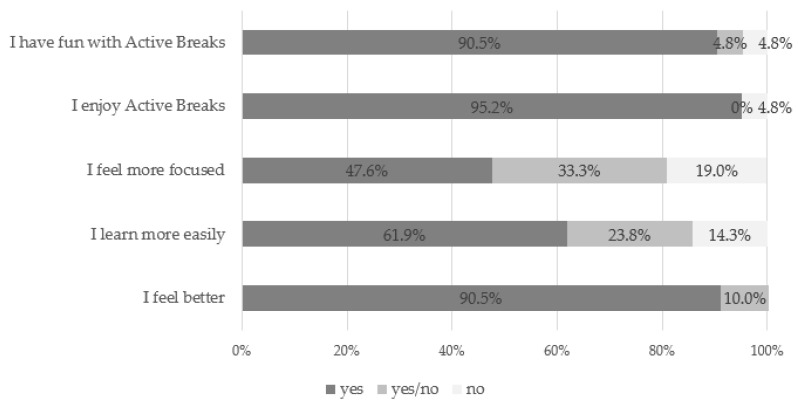
Children’s answers to the AB questionnaire.

Figure 3 shows the children’s feedback after the AB programme. Almost the entire sample reported feeling better after the intervention (90.5%), having fun with ABs (90.5%) and enjoying the AB intervention (95.2%). Almost half of the children (47.6%) felt more focused after the AB programme and 61.9% said they learned more easily.

**Figure 4:**
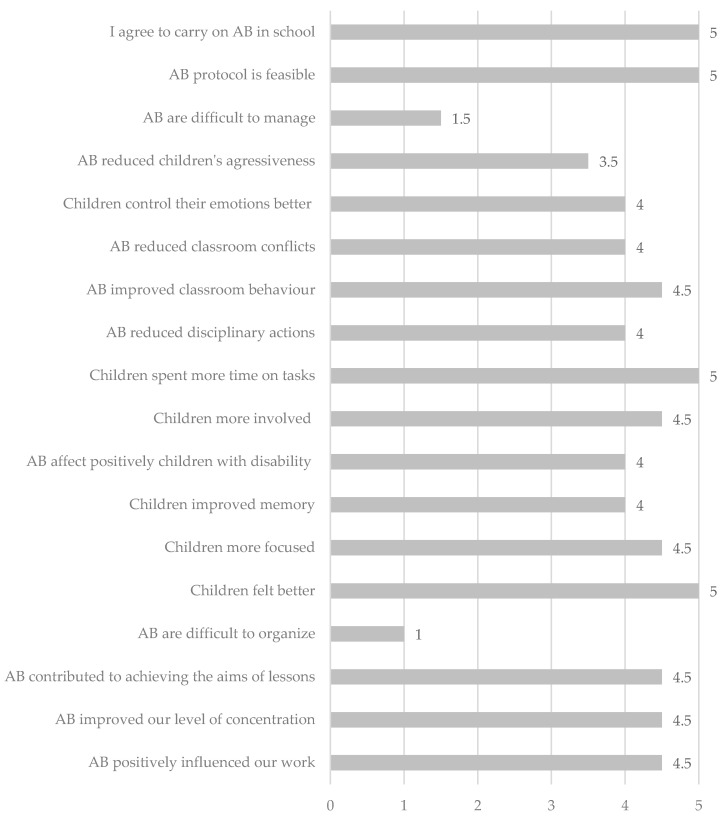
Teachers’ answers to the AB questionnaire.

Teachers declared that the AB group improved the time spent on tasks, classroom behaviour and involvement during work activity. Furthermore, teachers reported that the AB intervention reduced conflicts among children. They also reported a positive influence on their work. Teachers assigned positive scores regarding AB feasibility, effectiveness, and management, and they expressed their willingness to repeat the experience (Figure 4). Teachers and children in the control group did not fill out the questionnaire, because they did not perform the active breaks.

## 4. Discussion

This study evaluated the feasibility and effectiveness of an AB intervention, in terms of physical activity level. The AB group obtained a pre-post general improvement in all Actigraph accelerometer parameters. Moreover, after the end of the intervention, the children showed increased levels of MVPA and number of steps, with significant differences compared with the control group. Other studies showed that classroom-based ABs increased children’s physical activity levels [23,38,39]. Daily Smith et al. investigated the effect of ABs with academic content (active learning) on PA level, cognitive and academic outcomes. They found a slight improvement in children’s PA level and classroom behaviour, but no effect of ABs on academic achievement and cognitive function [40]. However, such a lack of cognitive effect of AB could be due to the investigated cognitive domain (mainly memory and long-term memory). On the contrary, it might be important to consider multisensory perception, that which is impaired in several developmental disorders, such as dyslexia [41], and autism [42]. A benefit of one bout of exercise on multisensory processing, (i.e., the ability to appropriately integrate information from different sensory modalities), has been demonstrated in the elderly [43], but there is also the need to better investigate this cognitive ability in children, as it represents a foundation to learning and cognitive development [44].

Therefore, a school-based intervention of active breaks of 10 min twice a day with HIIT exercises inside the AB protocol could allow one to reach the daily 60 min of MVPA recommended by the WHO and could represent a good strategy to promote active behaviours within the classroom context, encouraging good practice, to be translated into everyday life. Our results are in line with those reported by Drummy et al. [23]

This AB intervention also seems to be effective in children with WtHR ≥ 0.5, who increased the time spent in MVPA to the same extent as children with WtHR < 0.5. This finding is of particular relevance, considering that WtHR is an index of cardiovascular risk [34,35,36] in adulthood and children with high WHTR are those who most need to move actively. On the contrary, the children of the control group worsened their PA habits, and the level of time spent in MVPA at follow-up worsened to a greater extent in children at risk, compared with those not at risk.

Children expressed a positive evaluation toward ABs, claiming that they felt better, and more focused, and that they experienced enjoyment. Moreover, teachers highlighted the beneficial effects of Abs, in terms of general wellbeing, classroom behaviour and improvement in work activity. The feedback from both children and teachers confirms various aspects of the feasibility of AB programmes in the school context: for children, the AB programme was fun, pleasant and gave them a higher tolerance to being in the classroom; teachers found it manageable, useful for controlling children’s classroom behaviour and, in general, effective. Other studies showed that classroom-based ABs improved children’s time on task behaviour during academic instruction [38,39]. The favourable opinion of the teachers was also expressed with regards the feasibility of the AB intervention. Their satisfaction with the project was such that they spontaneously continued to propose the active breaks also in the following school year, outside the terms of the study. All this supports the possibility of including ABs within the normal school curriculum [25,38].

This study has some limitations. First of all, the sample was small and not randomized. It was very hard to randomize the groups, because few teachers were interested in participating. Other studies focused on classroom AB intervention confirmed these issues [39,45,46]. The collaboration of teachers is fundamental to carry out the project, because they are an active part of it, especially to develop a sustainable initiative that should continue when the research team no longer supervises the project. Erwin et al. showed that motivated teachers were essential to keep adherence to the project irrespective of teachers who did not really believe in the power of this instrument [45]. For this reason, we performed ABs only in the class in which the teachers were really interested to support this activity. Due to this limitation, the data of the questionnaire from teachers should be treated with caution and needs future verification. Another aspect that could represent a limitation is the seasonality, because the baseline assessments were made in early January (winter) while the follow-up in May (spring). Moreover, the other two aspects that could represent a limitation are the following: active breaks intensity has never been assessed during the intervention and physical activity has been evaluated only with accelerometers. Despite these limitations, this pilot and feasibility study was useful to test how to implement this type of intervention in the school setting. The results encourage us to implement the protocol with a structured study involving a larger sample of children and teachers, and investigating other aspects, in particular the effects of ABs on cognitive functions and quality of life [47,48].

To our knowledge, only one other study was conducted in Italy to evaluate the feasibility and effectiveness of classroom AB intervention [25]. The study of Calella et al. used different tools for assessing the PA levels and different ways of administering active breaks; however, it led to findings similar to our own. Unlike the study of Calella et al., we compared an intervention group with a control group, and measured the PA level at follow-up after the AB protocol was finished. Therefore, the observed improvements cannot be attributed to the time dedicated to ABs in the classroom, but to the spontaneous physical activity of the children, which also continued after the intervention. Other studies are needed to confirm these findings, assessing whether classroom active breaks can also affect children’s motor habits outside the school context. Notwithstanding its limitations, this study was useful to plan and manage a future study with a longer intervention period and a larger sample of children and teachers involved.

## 5. Conclusions

This AB protocol provided evidence of being effective and feasible, thanks to its simple application and sustainability, as confirmed by the positive feedback from both children and teachers. AB could be a possible intervention to reduce inactivity, contributing to reach the goal of 60 min daily MVPA. This pilot and feasibility study showed the feasibility and effectiveness of the AB protocol in primary school children and represents the basis for a future controlled trial.

## Figures and Tables

**Table 1:** Components of the Active Break Protocol.

Phase	Aim	Examples of the Type of Exercises	Duration
Warm-up	Physical activation and mobility	“The traffic light”: all children stand next to the desk and wait for the teacher’s commands. When the teacher says “green” the children have to start running quickly on the spot, when the command is “yellow” the children have to slow down and march on the spot; finally, when the teacher says “red” the children have to stop in position.	3 min
Tone-up	High-intensity interval training (HIIT); balance; cooperation; coordination exercises	HIIT “jumping jack”: all children perform jumping jack on the spot for 40”, followed by 20” of rest in balance position	5 min
Cool-down	Breath control and relaxation exercises to restart the usual academic lesson	“Flower and the candle”: children learn the correct way to breathe by imagining to inhale the scent of a flower and exhale while blowing on a candle	2 min

**Table 2:** Outcome measures at baseline, follow-up and changes at 14 weeks.

Variables	Active Break (AB) Group (*n*: 16)				Control Group (*n*: 12)				
	Baseline	Follow Up	Change	Within-Group	Baseline	Follow Up	Change	Within-Group	Between Groups
	Mean ± SD	Mean ± SD	Mean ± SD	*p* Value	Mean ± SD	Mean ± SD	Mean ± SD	*p* Value	*p* Value ^a^
Sedentary Activity (min/week)	8124.0 ± 485.3	7955.3 ± 435.2	−168.7 ± 504.0	0.20	7753.3 ± 432.8	7880.8 ± 548.8	+127.5 ± 609.7	0.48	0.79
Weekly MVPA (min)	292.0 ± 36.7	356.4 ± 171.4	+64.4 ± 136.0	0.07	300.5 ± 143.6	258.3 ± 98.8	−42.2 ± 103.5	0.19	0.03
Daily MVPA (min)	36.5 ± 18.4	44.6 ± 21.4	8.05 ± 17.0	0.08	42.2 ± 19.7	36.9 ± 14.1	−5.3 ± 14.4	0.22	0.06
MVPA%	2.9 ± 1.5	3.6 ± 1.7	0.7 ± 1.4	0.07	3.1 ± 1.4	2.7 ± 1.0	−0.4 ± 1.1	0.22	0.03
Step Counts (*n*/week)	43,921.4 ± 13,555.5	57,948.3 ± 14,401.8	14,026.9 ± 13,746.6	0.01	53,041.3 ± 17,089.7	47,602.3 ± 16,536.9	−55439.0 ± 14,076.3	0.21	0.01

^a^ Changes in measures between baseline and follow-up are compared using ANCOVA with correction for baseline scores.
